# Application of immersion pre-treatments and drying temperatures to improve the comprehensive quality of pineapple (*Ananas comosus*) slices

**DOI:** 10.1016/j.heliyon.2020.e05882

**Published:** 2021-01-01

**Authors:** Wahidu Zzaman, Rahul Biswas, Mohammad Afzal Hossain

**Affiliations:** Department of Food Engineering and Tea Technology, Shahjalal University of Science and Technology, Sylhet-3114, Bangladesh

**Keywords:** Pre-treatment, Hot air drying, Physicochemical properties, Rehydration capacity, Shrinkage evaluation, Color, Antioxidants

## Abstract

Drying of pineapple slices combined with different pre-treatments was done to reduce various adverse changes by adding satisfactory value. Process optimization was done by dipping the pineapple slices in four different solutions (1% trehalose, 2% NaCl, 10% sucrose, and 10% fructose) before drying. The effects of different pre-treatments and drying temperatures of 50, 55, and 60 °C with a constant 30% relative humidity (RH) were optimized based on the quality attributes, drying time and microbial load of dried pineapple slices. The optimal drying temperature was 55 °C using 1% trehalose pre-treatments based on the physical and biochemical properties. The reconstituted dried pineapples implied at this condition, contributed to the better structure preservation as indicated by the lower shrinkage (0.21) and the higher Coefficient of Rehydration (0.941), and rehydration ratio (6.840). On the other hand, the retention of color, vitamin C, B vitamins, and antioxidant activity of the samples were decreased by increasing drying time and temperatures. The highest Total Phenolic Content (121.02 mg GAE/100g), Total Flavonoid Content (8.72 mg QE/100g), and DPPH radical scavenging activity (7.22 EC_50_ g/100g) were found at 60 °C drying temperature with 10% fructose pretreatment's samples. The lowest drying time required was 7.64 h using 2% NaCl pre-treatment at 60 °C, considering the time required to reach 20% moisture content in the dried product at 30% RH. Based on the reported results, it is concluded that 1% trehalose at 50 °C can be used to develop high quality pineapple snacks, which maintained the maximum desired physicochemical and nutritious properties. This study could play an essential role in meeting the emerging demand of developing good quality nutritious dried pineapple snacks.

## 1 Introduction

Pineapple is one of the most desired and generally consumed succulent non-citrus fruits, mainly for its splendid sensorial characteristics and revitalizing sugar action. It has been considered as a vibrant source of the essential nutritients, such as minerals, vitamins (vitamin C, thiamine, vitamin B_6_, folate), antioxidants, dietary fiber, etc (Mohammed et al., 2020; Bartolomé et al., 1995; Ramallo and Mascheroni, 2012). The demand of pineapple for internal consumption and also for export is increasing day by day. But, due to the high moisture content of fresh pineapple, it has a lower shelf-life. Hence, a large amount of pineapple destruction results from inappropriate storage, lack of transportation, and unimproved marketing facilities. So, preservation of pineapple in some appropriate form is a demanding public concern (Hossain et al., 2020a). If it is possible to preserve the excess pineapples of peak season by ensuring the proper quality, they can be exported to other countries after meeting the country's off-season demand.

Drying has been considered as one of the most crucial preservation techniques, commonly applied for extending shelf-life of fruits and vegetables, and improving food stability by minimizing moisture content, chemical degradation, and microbial load (Brasiello et al., 2017). Unfortunately, the drying process can provoke countless physicochemical alterations in tissue structures that result in poor reconstitution properties in rehydrated products (Saifullah et al., 2019; Lewicki, 1998). This leads to physicochemical changes like chemical composition, color, texture, shrinkage, and nutritional variations, which have a high impact on its overall quality of dried products and, therefore, consumers' acceptability (Sasongko et al., 2020; Hossain et al., 2021). In this context, the drying of various foodstuffs combined with different pre-treatments and combinations has been proposed to diminish various negative changes by adding satisfactory value. There are different kinds of pre-treatments have been used, which include immersion in chemical solutions viz. salt, sugar, potassium carbonate, or organic acids (Adiletta et al., 2018a; Junqueira et al., 2017), blanching (Sarkar et al., 2021; Doymaz, 2010) and several physical pre-treatments (Adiletta et al., 2016a; Fratianni et al., 2018), etc. Among several pre-treatments, immersion in chemical solutions, which is also known as dipping pre-treatment or osmotic dehydration has been considered as the best technique to retain and improve physicochemical and nutritional qualities of dried fruits and vegetables in recent years (Adiletta et al., 2016a, 2018a; Brasiello et al., 2017). Osmotic dehydration is simply the process of removing unbound moisture by dipping a foodstuff in a hypertonic solution. Usually, the product is placed in a treatment tank with a hypertonic solution inside it. Several chemicals, like different kinds of sugars, sodium chloride (NaCl), and organic acids are used in this regard (Lewicki, 1998).

Pineapple being a very perishable commodity has gained the researchers’ attention to preserve it by developing a notorious dehydration system, in recent years. Consequently, several previous studies have been conducted on the immersion pre-treatment of pineapple slices prior to drying at different low temperatures. Ramolla et al. (2004), and Lombard et al. (2008) used sucrose pre-treatment to dry pineapple slices at 30–50 °C. Their studies claimed that immersion pre-treatment increased the solid gain and reduced the quality change in dried pineapple. Zapata et al. (2011) reported on the optimization of osmotic dehydration of pineapple using citric acid pre-treatment by response surface methodology at the temperature range of 25–45 °C. The immersion pre-treatment in potassium metabisulphite solution (0.25% w/v) was found to retain the better quality characteristics of dried pineapple slices in another study (Rani and Tripathy, 2019). Onal et al. (2019) found that dipping of apple slices in trehalose, sodium chloride and sucrose solution reduce the changes in color, shrinkage and other quality parameters after drying. However, to the best of our knowledge, very limited previous study was conducted on Giant Kew varities of pineapple using trehalose, sodium chloride, sucrose, and fructose pre-treatment and 55, 60, and 66 °C drying temperatures.

In this context, the primary purpose of this research work was to evaluate the impact of four different immersion pre-treatments, such as 1% trehalose, 2% sodium chloride, 10% sucrose and 10% fructose on the hot air drying of pineapple slices at three different temperatures, such as 50, 55, and 60 °C with a constant 30% relative humidity. Relative humidity was kept minimum to reduce the quality loss of pineapple slabs after dehydration as it reduces the drying time. The pre-treatments and drying temperatures are selected based on previous literatures (Önal et al., 2019; Adiletta et al., 2018a; Brasiello et al., 2017). The changes in physicochemical and nutritional properties (shrinkage evolution, rehydration capacity, color, antioxidant activity, β-carotene, vitamin B_1_, B_2_, B_3_ and B_5_, vitamin C, citric acid content, pH value, total soluble solids), and microbial load of dried pineapples were also assessed.

## 2 Materials and methods

### 2.1 Sample preparation

Freshly harvested pineapples of Giant Kew (locally known as kalendar) varieties were collected from the Citrus Research Institute, Jaintiapur, Sylhet, Bangladesh. The pineapples used in this study were grown under natural conditions during August–September, 2019. After harvesting, pineapples were brought into the laboratory and washed with clean water. Then, they were peeled and cored manually and cut into 1 ± 0.02 cm thickness slices. As the nutrient content is different between the top and base portions of the fruits (Ramallo and Mascheroni, 2012; Miller and Hall, 1953), the central segment of each pineapple was only used in experiments. The average initial moisture content of the fresh pineapple slices was 90.78% (w.b).

### 2.2 Pre-treatment process

The pineapple slices were immediately immersed into four different solutions (1% trehalose, 2% NaCl, 10% sucrose, and 10% fructose), at 25 °C for 30 min as described by Albanese et al. (2007) with slide moderations. Five types of samples were used in this study: untreated pineapple slices (UTR), 1% trehalose (1TR), 2% NaCl (2TR), 10% sucrose (3TR), and 10% fructose treated pineapple slices (4TR). The concentrations of different pre-treatment solutions are selected based on the trial experiments and previous literatures (Önal et al., 2019; Adiletta et al., 2018a; Brasiello et al., 2017).

### 2.3 Drying process

**Figure 1:**
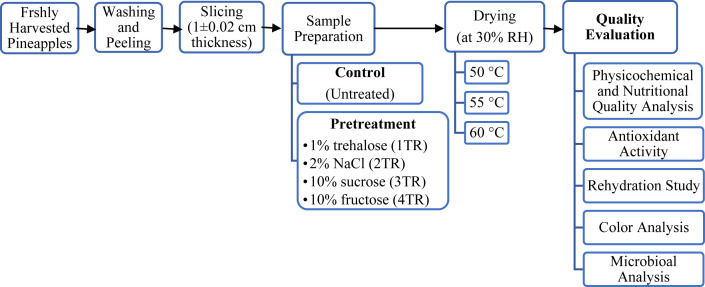
Schematic diagram of the research design.

After pre-treatment, both of the treated and untreated pineapple slices were placed in a dryer tray. The drying operation was carried out at three different temperatures- 50, 55, and 60 °C in a constant 30% relative humidity using a Humidity Chamber (Model: VS-8111H-150, input 220V 50Hz 16A 1 phase, Vision Scientific Co. Ltd., South Korea). The relative humidity was maintained 30% to reduce the drying time and get the desired nutritional and physical quality characteristics of the dried pineapple slabs. The weight changes were recorded until the difference between two consecutive weights was about 0.01g. The total experimental protocol is shown in Figure 1.

### 2.4 Physicochemical and nutritional quality analysis

#### 2.4.1 Total soluble solid (TSS)

A hand refractometer (Model: MASTER-AGRI, ATAGO, Japan) was used to measure the total soluble solid, and the data were recorded as °Brix. The real TSS content of dried samples was estimated by multiplicating each reading with the dilution factor (Abeywickrama et al., 2004).

#### 2.4.2 pH

The fresh and dried samples were made into juice. Then pH was measured using a digital pH meter (Model: HI-2211, Hanna Instrument, USA) (A.O.A.C., 2000).

#### 2.4.3 Percentage of titratable acidity (TA)

Titratable acidity was expressed as percentage citric acid (Abeywickrama et al., 2004), and the value were calculated from the following Eq. (1):

(1) $$\%\;Citric\;Acid\;\left(mg/100g\right)=V\mathrm{olume of NaOH}\times \mathrm{Dilution factor}\times \mathrm{Citric acid factor}\times 100$$

Here, citric acid factor is 64 mg.

#### 2.4.4 Determination of β-Carotene

The β-Carotene content was analyzed spectrophotometrically at 452 nm using a UV–Vis spectrophotometer (Model-T60U, PG instruments limited, UK) for total carotenoids (Rodriguez-Amaya, 2001). All analyses were done for triplicates and the average results were expressed as mg/100g using calibration curves (y = 0.5442x + 0.0093; R^2^ = 0.9998) of β-carotene as standard.

#### 2.4.5 Determination of vitamin B

The vitamin B content of fresh and dried pineapple samples was also measured by modified spectrophotometric methods of Fernandes et al. (2015); and Fernandes et al. (2016). Absorbance readings were carried out using a UV–Vis spectrophotometer (Model-T60U, PG instruments limited, UK) at 254 nm (B_1_), 320 nm (B_2_), 265 nm (B_3_), and 215 nm (B_5_). Calibration curves were prepared using thiamine hydrochloride (B_1_) (y = 44.526x + 0.0051; R^2^ = 0.9994), riboflavine (B_2_) (y = 23.8x + 0.0213; R^2^ = 0.9998), nicotidamide (B_3_) (y = 42.173x + 0.0613; R^2^ = 0.9994), and d-pantothenic acid hemicalcium (B_5_) (y = 29.608x + 0.0723; R^2^ = 0.9993).

#### 2.4.6 Determination of ascorbic acid (vitamin C)

The determination of Vitamin C content in fresh and dried pineapple slices was examined by a modified method described by Salkic et al. (2009). Absorbance readings were taken by a UV–Vis spectrophotometer (Model-T60U, PG instruments limited, UK) at 266 nm at room temperature, using 0.056 M sodium oxalate solution as blank. L-ascorbic acid was used as a standard to prepare calibration curve (y = 10.257x + 0.0289; R^2^ = 0.9958).

#### 2.4.7 Antioxidant activity

The potential antioxidant activity was measured by employing the Total Phenolic Content (TPC), Total Flavonoid Content (TFC), and 2,2-diphenyl-2-picryl-hydrazyl (DPPH) radical scavenging assay method. For that purpose, extracts of fresh and dried samples were prepared, succeeding the methodology proposed by Rahman et al. (2016) and Eim et al. (2013).

##### 2.4.7.1 Determination of total phenolic content (TPC)

Folin-Ciocalteu method was used to determine the total phenolic content in the extracts of dried pineapple, as described by Marquele et al. (2005). Absorbance readings were carried out using a UV–Vis spectrophotometer (Model-T60U, PG instruments limited, UK). Here, supernatant was measured at 765 nm against a reagent blank. Gallic acid standard curve (y = 0.9028x + 0.054; R^2^ = 0.9998) was used to estimate the total phenolic content in the extract, which was expressed as gallic acid equivalents (mg GAE/100g).

##### 2.4.7.2 Determination of total flavonoid content (TFC)

The total flavonoid content of pineapple sample extract was determined by the aluminum chloride spectrophotometric assay (Chang et al., 2002). Absorbance readings were carried out by a UV–Vis spectrophotometer (Model-T60U, PG instruments limited, UK) and measured at 510 nm against a reagent blank. The total flavonoid content was calculated from a calibration curve (y = 0.51x + 0.0268; R^2^ = 0.9941), and the result was expressed as quercetin equivalent (mg QE/100g) for the flavonoid contents.

##### 2.4.7.3 Determination DPPH radical scavenging activity

The antioxidant activity of the pineapple extract was determined by the 2,2-diphenyl-2-picryl-hydrazyl (DPPH) radical scavenging assay (Adiletta et al., 2018b) using UV-Vis spectrophotometer (Model-T60U, PG instruments limited, UK) at 517 nm at 25 °C (A_sample_). A solution without the extract was used as a reagent blank (A_control_).

The ability of the sample to scavenge DPPH radical was calculated by the following Eq. (2):

(2) $$\%\;DPPH\;radical\;scavenging\;activity=\frac{Abs_{control}-Abs_{sample}}{Abs_{control}}\times 100\;$$

where, Abs_control_ = the absorbance of blank; Abs_sample_ = the absorbance of the sample.

The free radical scavenging activity, determined by DPPH, was expressed as EC_50_ value. A graph of the extract concentration (g/100g) versus antioxidant capacity (%) were used to determine EC_50_ value.

#### 2.4.8 Rehydration study

Rehydration study of the dried pineapple slices was executed for 2 h at 25 °C. Rehydration ratio (RR) is the most crucial foundation to form a base material for additional utilization. The rehydration ratio reveals the novelty gained and the aptness attribute of a dried product as calculated by Eq. (3) (Ranganna, 1986).

(3) $$Rehydration\;Ratio=\frac{Weight\;of\;the\;Soaked\;Product\;\left(W_{r}\right)}{Weight\;of\;Dehydration\;Product\;\left(W_{d}\right)}$$

The weights of the samples were taken using an analytical balance (Model: FA2004B, Bio-Equip, China) with readability of 0.0001 g. The coefficient of rehydration (COP), indicates the degree of weight recovery to the fresh product. It is estimated according to Eq. (4) as described by Khraisheh et al. (2004):

(4) $$\mathrm{Coefficient of}Rehydration=\frac{W_{r}\left(100-X_{0}\right)}{W_{d}\left(100-X_{d}\right)}$$

where, W_r_ = weight of the soaked product, W_d_ = weight of the dehydration product, X_0_ = moisture content (% wet basis) of samples before drying, X_d_ = moisture content (% wet basis) of the sample after drying.

#### 2.4.9 Surface color measurement

Surface colors of pineapple slices were determined through a colorimeter (Model No: PCE-CSM4, PCE instruments, Germany). A white ceramic plate was used to standardize the apparatus before the measurements. To analyze the color changes between fresh and dried samples, color values (L∗, a∗, and b∗) were measured. For fresh specimens, an average of three independent values of pineapple slices from different zones was taken. The Hue angle and Chromaparameters were estimated from those values using the following Eqs. 5(a), 5(b), and (6), respectively (Ramallo and Mascheroni, 2012):

5(a) $$Hue\;Angle=tan^{-1}\frac{b}{a}\;,\;\left(when\;a\;>\;0\right)\;$$

5(b) $$Hue\;Angle=180+tan^{-1}\frac{b}{a}\;,\;\left(when\;a\;<0\right)$$

(6) $$Chroma=\sqrt{a^{2}+b^{2}}$$

The white index (WI) of pineapple samples were also calculated. It indicates the amount of whiteness of food samples. The WI was estimated using Eq. (7) (Adiletta et al., 2016b):

(7) $$\mathrm{WI}=100-\sqrt{{\left(100-L^{*}\right)}^{2}+{\left(a^{*}\right)}^{2}+{\left(b^{*}\right)}^{2}}$$

Browning index differences (ΔBI) was calculated by Eq. (8) (Pothula et al., 2015):

(8) $$\Delta BI=BI-BI_{0}$$

where, $BI=\frac{100}{0.17}\left(\frac{a^{*}+1.75L^{*}}{5.645L^{*}+a^{*}-3.012b^{*}}-0.31\right)$, BI_0_ = the initial BI value of pineapple samples.

Total color difference (ΔE) was determined by Eq. (9) (Vega-Gálvez et al., 2012)

(9) $$\Delta E=100-\sqrt{{\left(L^{*}-L_{0}^{*}\right)}^{2}+{\left(a^{*}-a_{0}^{*}\right)}^{2}+{\left(b^{*}-b_{0}^{*}\right)}^{2}}$$

where, L_0_∗ = the initial L∗ value of the samples, a_0_∗ = the initial a∗ value of the samples, b_0_∗ = the initial b∗ value of the samples.

#### 2.4.10 Shrinkage evaluation

The shrinkage evaluation was conducted in terms of the mean ratio of dried sample volume with moisture content X, V_b_(X) to the sample volume within initial moisture content V_b,0_ as described by Eq. (10) (Russo et al., 2019).

(10) $$S_{b}=\frac{V_{b}\left(X\right)}{V_{b,0}}$$

where, S_b_ = volumetric shrinkage coefficient, V_b_(X) = sample volume with moisture content X, V_b,0_ = sample volume within initial moisture content.

#### 2.4.11 Determination of microbial load (fungal and bacterial)

Microbial load analyses of the dried samples were performed for bacterial and fungal load determination. For fungal load, Mortin Rose Bengal Agar (Oxoid, England) was used. Approximately 18.0 g agar was dissolved in 1000 mL saline (0.89% NaCl) water. For bacterial load, standard plate count agar (HiMedia, India) [18.0 g agar was dissolved in 1000 mL of saline water (0.89% NaCl)] were used. In a test tube, 1.0 g of the sample was mixed, where 9 mL of autoclaved saline (0.89% NaCl) water poured previously. Serial dilution technique was used for both fungal and bacterial load determination. One mL of diluted solutions made from different samples was poured in each Petri dish, where 20–25 mL of the prepared media were previously poured and cooled for solidification. The Petri dishes were circumvolved for better mixing. The plates were then placed in an incubator (Thermo Fisher Scientific Inc., Model-IGS60) at 37 and 30 °C for bacteria and fungi, respectively. The incubated plates were examined after 24 and 72hr for bacteria and fungi, respectively, and a digital colony counter (BEXCO 220V) was used for counting the colonies (A.O.A.C., 2000).

#### 2.4.12 Determination of drying time

The drying time was determined based on the time required to reach 20% product moisture (wet basis). The weight changes were recorded during drying until the difference between two consecutive weights was about 0.01 g. However, as different samples’ endpoints of drying had a different moisture content (%), a standard 20% moisture content was considered for comparison (Önal et al., 2019). Eq. (11)was used to calculate moisture content (Ranganna, 1986):

(11) $$Moisture\;Content\;\left(MC\right)\left(wet\;basis\right)=\frac{W_{1}-W_{2}}{W_{1}}\times 100$$

where, MC = moisture content of the sample (% w.b.), W_1_ = weight of the sample before drying (g), W_2_ = weight of the sample after drying (g).

The drying time was estimated by taking the differences of the time required to reach 20% moisture content and initial time and calculated in hour (hr).

### 2.5 Statistical analysis

All the results were reported as the mean ± standard deviation (SD) using the well-known Software package Minitab (version 19.2020.1). Analysis of variance (ANOVA) and Tukey's test was applied for mean comparison. The least significant difference was calculated for the significant data at P < 0.05.

## 3 Results and discussions

### 3.1 Total soluble solids, total acid, and pH

Total Soluble Solids of dried pineapple samples were higher than the fresh pineapple (12 ± 0.1 °Brix) as the sugar and other solid contents become concentrated at the dried pineapple samples. The highest TSS value was 89.33 ± 3.06 °Brix using sucrose pre-treatment, whereas the lowest value was 76.67 ± 3.06 °Brix for untreated sample dried at 60 °C (Table 1). Zhao et al. (2017) reported similar observations that there was an increase in total soluble solids of osmotically dehydrated frozen mango cuboids. They found that when mango samples were immersed in a concentrated sugar solution, the final TSS increased. Our findings support the study of Expedito et al. (1996) who found that TSS content of osmotically dehydrated pineapple cubes increased substantially.

**Table 1:** Total Soluble Solids (TSS), Titratable Acidity (TA) and pH value in untreated and pre-treated dried pineapple slices.

Sample Condition		Total Soluble Solids (TSS) ^o^Brix	Titratable Acidity (TA) (mg/100g)	pH
Fresh		12 ± 0.10^f^	691.34 ± 85.63^a^	3.44 ± 0.01^i^
Drying at 50 °C	UTR	80.00± 2.00^cde^	378.93 ± 13.48^b^	4.22 ± 0.02^a^
	1TR	82.66 ± 5.03^bcd^	209.46 ± 5.11^f^	4.13 ± 0.02^b^
	2TR	82.00± 2.00^bcde^	267.75 ± 6.38^de^	3.92 ± 0.02^c^
	3TR	89.33 ± 3.06^a^	195.19± 6.51^f^	4.10 ± 0.10^b^
	4TR	85.00± 7.07^ab^	206.04 ± 6.24^f^	3.92 ± 0.01^c^
Drying at 55 °C	UTR	80.00± 2.00^cde^	380.93 ± 8.49^b^	3.73 ± 0.02^e^
	1TR	80.00± 2.00^cde^	254.75 ± 6.53^e^	3.69 ± 0.01^e^
	2TR	82.00± 2.00^bcde^	273.39 ± 5.42^de^	3.69 ± 0.02^e^
	3TR	84.00± 2.00^bc^	256.41 ± 6.11^e^	3.59 ± 0.02^f^
	4TR	82.67 ± 4.16^bcd^	214.29 ± 6.94^f^	3.81 ± 0.02^d^
Drying at 60 °C	UTR	76.67 ± 3.06^e^	390.12 ± 8.26^b^	3.53 ± 0.01^g^
	1TR	80.67 ± 3.06^bcde^	265.45 ± 5.53^de^	3.49 ± 0.01^gh^
	2TR	78.00± 2.00^de^	301.35 ± 6.03^cd^	3.36 ± 0.01^j^
	3TR	83.33 ± 3.06^bcd^	283.79 ± 6.92^de^	3.49 ± 0.01^ghi^
	4TR	80.67 ± 3.06^bcde^	327.92 ± 11.56^c^	3.47 ± 0.01^hi^

-All the values in the table are mean ± SD of three independent determinations. Samples in the same column with different superscript letters differ significantly at p < 0.05.

Titratable acidity (TA) of dried pineapple samples was decreased in contrast with the treated samples. The highest value of titratable acidity was 390.932 ± 8.499 (mg/100g) for the untreated samples dried at 60 °C temperature. The dried pineapple samples' pH values were higher than the fresh sample as total acidity was reduced by pre-treatment. The pH value of fresh pineapple was 3.44 ± 0.01, and the pH value of dried pineapple samples ranged from 3.36 ± 0.01 to 4.22 ± 0.02. Both TA and pH of about all pineapple treatments were statistically significant at different temperatures (Table 1). The titratable acidity was increased with the rising drying temperature. Usually, the pH exhibited a declining trend with increasing temperature. Obadina et al. (2018) has also reported this reversely sequenced observation. Table 1 exhibits TSS, TA, and pH of different pineapple samples.

### 3.2 β-Carotene, B vitamins and ascorbic acid (vitamin C)

The β-carotene is considered as a vital provitamin A found in pineapple. The amount of β-carotene in the fresh pineapple sample was 87.65± 1.84μg/100g (Table 2). It reduced in the dried samples as compared to the fresh samples. Among different treated samples, fructose pre-treated samples showed the highest for retaining β-carotene. These values were decreased slightly with increasing drying temperature. The loss of β-carotene estimated as 11.04 ± 0.98, 12.19 ± 2.57 and 13.81 ± 1.13 (%), at 50, 55, and 60 °C drying temperature, respectively for fructose pre-treated dried samples. Overall, dried pineapple samples observed to restore a higher percentage of β-carotene than the fresh pineapple. Usually, more β-carotene retention were found at higher concentrations of pre-treatment solutions. It is thought that, the pre-treatment solutions formed an oxygen barrier on the cell surface, which may delay β-carotene oxidation. A higher percentage of β-carotene restored at higher concentrated solutions were indicated by Song et al. (2018), and Lago-Vanzela et al. (2013). Similar results were also found by Fernandes et al. (2015), where they showed that the amount of β-carotene was slightly decreased during the drying process. The high temperature stimulates the transition of double bonds that results in the decreasing carotenoid as indicated by brightening of the color (Meléndez-Martínez et al., 2010; Fratianni et al., 2010).

**Table 2:** Amount of β-Carotene, Vitamin B_1_, B_2,_ B_3_, B_5_ and Ascorbic Acid (Vitamin C) in untreated and pre-treated dried pineapple slices.

Sample Condition		β-Carotene (μg/100g)	Vitamin B_1_ (mg/100g)	Vitamin B_2_ (mg/100g)	Vitamin B_3_ (mg/100g)	Vitamin B_5_ (mg/100g)	Ascorbic Acid (Vitamin C) (mg/100g)
Fresh		87.65 ± 1.84^a^	1.12 ± 0.07^a^	1.98 ± 0.03^a^	1.22 ± 0.02^a^	2.10 ± 0.02^a^	140.92 ± 0.44^a^
Drying at 50 °C	UTR	68.12 ± 0.44^gh^	1.05 ± 0.02^b^	1.06± 0.02^b^	1.09 ± 0.02^b^	1.96 ± 0.07^b^	69.61 ± 0.09^b^
	1TR	69.12± 0.25^fgh^	0.69 ± 0.01^k^	0.88 ± 0.02^d^	0.73 ± 0.01^i^	1.33 ± 0.02^h^	62.83 ± 0.09^d^
	2TR	71.68 ± 0.31^e^	0.74 ± 0.01^j^	0.83 ± 0.02^e^	0.79 ± 0.01^gh^	1.48 ± 0.02^g^	51.74 ± 0.09^g^
	3TR	75.42 ± 0.43^cd^	0.86 ± 0.01^e^	0.78 ± 0.02^g^	0.88 ± 0.02^d^	1.63 ± 0.03^d^	65.31 ± 0.43^c^
	4TR	77.96 ± 0.79^b^	0.78 ± 0.01^h^	0.67 ± 0.02^h^	0.80 ± 0.01^g^	1.50 ± 0.02^g^	49.86 ± 0.05^h^
Drying at 55 °C	UTR	67.20 ± 0.82^h^	0.93 ± 0.01^c^	1.01 ± 0.02^c^	0.97 ± 0.02^c^	1.86 ± 0.03^c^	62.79 ± 0.11^d^
	1TR	67.19 ± 0.74^h^	0.86 ± 0.01^e^	0.67 ± 0.01^h^	0.87 ± 0.01^de^	1.61 ± 0.02^de^	51.85 ± 0.08^g^
	2TR	70.97 ± 0.62^ef^	0.64 ± 0.01^l^	0.48 ± 0.01^k^	0.65 ± 0.01^j^	1.25 ± 0.02^i^	40.08 ± 0.07^l^
	3TR	74.69 ± 0.29^d^	0.83 ± 0.01^f^	0.68 ± 0.02^h^	0.86 ± 0.01^e^	1.57 ± 0.01^ef^	53.73 ± 0.13^f^
	4TR	76.93 ± 0.89^bc^	0.79 ± 0.01^g^	0.68 ± 0.03^h^	0.82 ± 0.01^f^	1.57 ± 0.02^ef^	45.53 ± 0.06^j^
Drying at 60 °C	UTR	60.99 ± 0.52^j^	0.91 ± 0.01^d^	0.81 ± 0.04^f^	0.98 ± 0.02^c^	1.87 ± 0.03^c^	60.96 ± 0.11^e^
	1TR	64.51 ± 0.71^i^	0.53 ± 0.01^n^	0.49 ± 0.02^k^	0.55 ± 0.01^k^	1.20 ± 0.02^j^	44.02 ± 0.12^k^
	2TR	69.55 ± 0.39^efg^	0.76 ± 0.01^i^	0.68 ± 0.03^h^	0.80 ± 0.01^fg^	1.49 ± 0.02^g^	36.06 ± 0.10^n^
	3TR	73.89 ± 0.13^d^	0.62 ± 0.01^m^	0.52 ± 0.04^j^	0.65 ± 0.01^j^	1.33 ± 0.01^h^	49.45 ± 0.09^i^
	4TR	75.53 ± 0.60^cd^	0.73 ± 0.01^j^	0.64 ± 0.03^i^	0.77 ± 0.02^h^	1.53 ± 0.03^fg^	39.04 ± 0.47^m^

-All the values in the table are mean ± SD of three independent determinations. Samples in the same column with different superscript letters differ significantly p < 0.05.

Pineapples are vital sources of water-soluble vitamin B (Bartolomé et al., 1995; Kennedy, 2014). Since dipping in solution was the primary therapy in this study, so it was vital to know whether any vitamin B has been lost during this pre-treatment process. After drying, the amounts of vitamin B_1_, B_2_, B_3,_ and B_5_ were higher in untreated dried samples than treated drying samples. The loss of vitamins B_1_, B_2_, B_3,_ and B_5_ may be occurred during the pre-treatments process due to leaching out to salt/sugar solutions. Vitamin B_1_ (Thiamin) is considered as the most heat-sensitive B vitamins. Its degradation follows a first-order kinetic reaction (Mauri et al., 1989). In the fresh sample, the amount of vitamin B_1_ was 1.12 ± 0.07 mg/100g. However, it reduced with the drying temperature and pre-treatments and varied from 0.53 ± 0.01 to 1.05 ± 0.02 mg/100g. Most of the samples differed significantly from each other. Vitamin B_2_ (Riboflavin) also may leach out in the treated samples as indicated by the lower value of the pre-treated samples in contrast with the untreated samples. These values were reduced gradually with increasing temperature. Fernandes et al. (2016); and Sheraz et al. (2014) found that vitamin B_2_ (Riboflavin) is light and temperature-sensitive product during food processing, which supports our current findings. Our study found that vitamin B_3_ reduced slightly in the treated samples than the untreated ones. However, they were decreased slowly with high temperature, which justifies the findings of Ball (2005). Vitamin B_3_ (Niacin) seems to be a very stable B vitamin, and it is slightly influenced by heat, light, and air (Ball, 2005). Pineapple is a good source of Vitamin B_5_ (Pantothenic acid), and it is widely distributed as free form, bound in coenzyme A and acyl carrier protein (Ball, 2005). The study also found that the retention rate of vitamin B_5_ during soaking pre-treatments and higher temperatures was the highest among other B vitamins. The quantity of vitamin B_5_ in the fresh sample was 2.10 ± 0.02 mg/100g, and in dried samples it ranged from 1.20 ± 0.02 to 1.96 ± 0.07 mg/100g. Hoppner and Lampi. (1993) also found that when foodstuffs were submitted to immersion pre-treatments before drying, the retention rate of vitamin B_5_ was higher. These values of vitamin B_1_, B_2_, B_3,_ and B_5_ of untreated and treated pineapple samples are shown in Table 2.

Vitamin C (ascorbic acid) is a temperature-sensitive and water-soluble vitamin as well. In the fresh pineapple, the vitamin C content was 140.92± 0.44 (mg/100g) (Table 2). The highest ascorbic acid retention 49.39 ± 0.09 (%) was in the untreated dried sample at 50 °C drying temperature, which implies that soaking and higher temperatures negatively affect vitamin C. The maximum loss of vitamin C observed at 60 °C drying temperature. The treated dried samples showed the lower value of vitamin C, which might be due to leaching out of ascorbic acid during pre-treatments as vitamin C is a water-soluble nutrient. Higher drying temperatures mostly degraded the value of vitamin C. Our findings support the previous studies, where researchers suggested that vitamin C degradation is greatly moisture and heat dependent (Ramallo and Mascheroni, 2012; Santos and Siliva, 2008). The ascorbic acid retention by untreated pineapples was higher than the treated samples. Several previous studies claimed that the reduction in vitamin C observed during drying indicates the instability of ascorbic acid at elevated temperatures (Santos and Siliva, 2008; Osundahunsi, 2008). However, the sucrose (osmosis) pre-treatment showed much protective effect on the ascorbic acid loss during drying. PardioSedas et al. (1994) also found that ascorbic acid was degraded in pineapple during storage under fixed temperature.

### 3.3 Antioxidant activity

**Figure 2:**
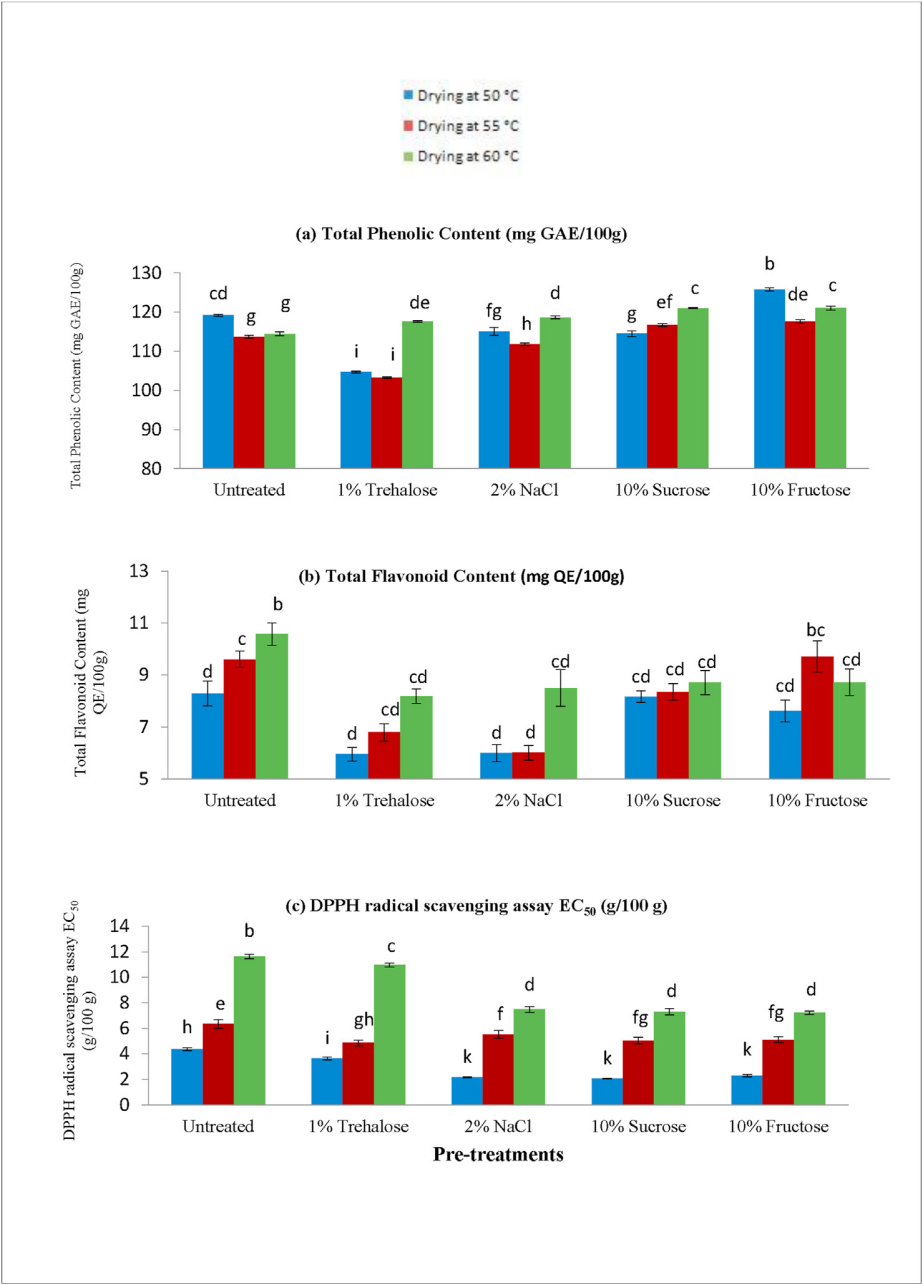
(a) Total Phenolic Content (TPC), (b) Total Flavonoid Content (TFC), and (c) DPPH radical scavenging assay values in untreated and pre-treated dried pineapple slices.

The antioxidant potential of the (a) Total Phenolic Content (TPC), (b) Total Flavonoid Content (TFC), and (c) DPPH radical scavenging assay values are shown in Figure 2.

The amount of TPC was 183.13 ± 1.69 (mg/100g) in the fresh pineapple samples, and after drying of the pineapple samples, the loss of the TPC ranged from 31.35 ± 0.42 to 43.59 ± 0.43 (%). Sucrose and fructose pre-treated samples showed better TPC retention than others. Initially, the TPC values decreased with increasing temperature. Vega-Gálvez et al. (2012) found that TPC was degraded with increasing drying temperature in dried apples. In this study, the TPC was initially reduced from 50 to 55 °C drying temperature; then again, it was increased at 60 °C. This might be due to the lower dehydration time required by the higher drying temperature. Thus, there was a short time for TPC degradation. Rodriguez et al. (2014) found lower TPC at higher dehydration temperature, which supports the findings of our study.

Several mechanisms could be explained for TPC loss at higher temperatures. Mendez-Lagunas et al. (2017) demonstrated the breakdown of lignin, thermal deterioration of phenolics, and the release of bound phenolic compounds as the possible pathways of TPC loss during drying. Horuz et al. (2017) suggested that the drying temperatures, different drying methods, and pre-treatments negatively influence the stability of total phenolic content in food. From our study, it was found that the proposed pre-treatments conserved the TPC content. Gumusay et al. (2015) also commented that some phenolic compounds usually bind with the protein, which may be a reason for declining TPC in dried samples as proteins are denatured at high temperatures.

The Total Flavonoid Content (TFC) of fresh pineapple sample was 45.49 ± 3.92 (mg/100g). The TFC value of dried samples varied from 5.95 ± 0.26 to 14.17 ± 0.43 (mg/100g). The loss of TFC ranged from 68.86 ± 1.77 to 86.93 ± 0.59 (%) after drying. Flavonoids are heat-sensitive compounds exhibiting antioxidant activity. The breakdown of flavonoids at higher temperatures could be a reason for reducing the total flavonoid at higher temperatures. As the temperature increased, it causes the degradation of cell walls resulting in the liberation of oxidative and hydrolytic enzymes, which can destroy the antioxidants in foods (Dewanto et al., 2002). The present study found that total flavonoids increased with increasing temperature in some cases, which is somewhat unstable with different treatments. These results can be explained by the shorter times required by higher drying temperatures; thus the pineapple slices had shorter exposure to heat, which resulted in a minor breakdown of TFC (Saifullah et al., 2019). Our findings are quite consistent with the summary of Roshanak et al. (2015), who suggested that total flavonoids were increased after heating at a certain temperature for a considerable time.

The EC_50_ value is the required sample concentration (g/100g) to hamper 50% of the DPPH radical scavenging activity. The EC_50_ value of fresh pineapple was 18.36 g/100 g, and the lowest amount found in the dried samples that are meaning the much higher antioxidant activity of dried samples than the fresh sample. The antioxidant activity was better retained (the lowest EC_50_ values) at lower drying temperatures than at higher drying temperatures. The reduction of antioxidant activity at higher temperatures were also supported by Hossain et al. (2020b) for moringa leaves, Adiletta et al. (2016a) for grape, Vega-Gálvez et al. (2012) for apple, and İzli (2017) for dried dates. However, the dipping pre-treatments were found to exhibit better retention of antioxidant activity of pineapple slices. Nora et al. (2014) claimed that partially oxidized polyphenols have higher antioxidant activity, which might be a reason behind the higher antioxidant activity of dried plant products.

### 3.4 Rehydration and shrinkage

The water uptake capacity of dried product during rehydration can be appraised by the Coefficient of Rehydration (COR) and Rehydration Ratio (RR) indexes. The amount and rate of water absorption during the reconstitution are crucial points that affect the quality of dried products. The physicochemical changes during drying are affected by the quality indicators of rehydration (Horuz et al., 2017; Lewicki, 1998). The coefficient of Rehydration (COR) and Rehydration Ratio (RR) values for pineapple samples were measured after 2 h of rehydration, as presented in Table 4. On average, a higher value of the coefficient of rehydration observed for pre-treated samples dried at 55 °C, which indicated a more exceptional ability of water absorption in pineapple samples (Table 3). Previous studies reported that the drying condition and physicochemical properties of a dried product affected the rehydration characteristics (Krokida and Marinos-Kouris, 2003; Khraisheh et al., 2004). Pineapple slabs dried at 55 °C using 1% trehalose solution showed the highest (6.840 ± 0.0204) rehydration ratio. Dried samples with dipping pre-treatment showed better rehydration capacity. Önal et al. (2019) and Atarés et al. (2008) found that osmotically dehydrated apple cylinders exhibited better solute retention during rehydration. Several previous researchers claimed that immersion in concentrated sugar solution has a protective impact on cell walls of different fruits and vegetables (Adiletta et al., 2016b; Betoret et al., 2015; Albanese et al., 2007; Aktaş et al., 2007).

**Table 3:** Coefficient of Rehydration (COR), Rehydration Ratio (RR) and Volumetric shrinkage coefficient (S_b_) in untreated and pre-treated dried pineapple slices.

Sample Condition		Coefficient of Rehydration (COR)	Rehydration Ratio (RR)	Volumetric Shrinkage Coefficient (S_b_)
Drying at 50 °C	UTR	0.486 ± 0.0008^l^	3.705 ± 0.0061^l^	0.22 ± 0.08^a^
	1TR	0.571 ±0.0028^k^	4.012 ± 0.0200^j^	0.22 ± 0.02^a^
	2TR	0.587 ±0.0029^j^	3.716 ± 0.0187^l^	0.30 ± 0.09^a^
	3TR	0.738 ±0.0052^g^	3.942 ± 0.0277^k^	0.31 ± 0.03^a^
	4TR	0.743 ±0.0053^g^	3.337 ± 0.0237^m^	0.32 ± 0.01^a^
Drying at 55 °C	UTR	0.842 ±0.0016^d^	6.504 ± 0.0123^b^	0.21± 0.13^a^
	1TR	0.941 ±0.0028^b^	6.840 ± 0.0204^a^	0.21 ± 0.07^a^
	2TR	0.704 ±0.0039^h^	4.574 ± 0.0253^g^	0.22 ± 0.05^a^
	3TR	0.911 ±0.0029^c^	5.039 ± 0.0162^e^	0.22 ± 0.03^a^
	4TR	0.939 ±0.0038^b^	4.388 ± 0.0176^h^	0.26 ± 0.03^a^
Drying at 60 °C	UTR	0.739 ±0.0009^g^	5.675 ± 0.0069^d^	0.21± 0.10^a^
	1TR	0.804 ±0.0028^f^	5.892 ±0.0208^c^	0.22 ± 0.08^a^
	2TR	0.649±0.0034^i^	4.156 ± 0.0218^i^	0.25 ± 0.05^a^
	3TR	0.828 ±0.0032^e^	4.546 ± 0.0176^g^	0.23 ± 0.02^a^
	4TR	1.039 ±0.0034^a^	4.680 ±0.0153^f^	0.22 ± 0.04^a^

-All the values in the table are mean ± SD of three independent determinations. Samples in the same column with different superscript letters differ significantly p < 0.05.

The most visible physical change occurs in dried fruits and vegetables is shrinkage. Several factors work behind shrinkage, negatively affecting the products' sensorial appeal (Mayor and Sereno, 2004; Brasiello et al., 2013). In the present study, the effects of pre-treatment and different air-drying temperatures (50, 55, and 60 °C) on shrinkage of pineapple slices were studied, and the changes in volume ratio as volumetric shrinkage coefficient (S_b_) were investigated. After drying, treated pineapple slices were less shrunk than the untreated one at 55 and 60 °C temperatures. So, it can be decided that different pre-treatments worked against the shrinkage of pineapple slices. These inspections are in good concurrence with previous results reported for apples (Önal et al., 2019; Moreira et al., 2000), gooseberry (Junqueira et al., 2017), and grapes (Adiletta et al., 2016a). Table 4 illustrates the coefficient of rehydration (COR), rehydration ratio (RR), and volumetric shrinkage coefficient (S_b_) in untreated and pre-treated dried pineapple samples.

**Table 4:** Color Parameters and White Index (WI), Browning Index Differences (Δ*BI*) and Total Color Differences (Δ*E*) of fresh, untreated and pre-treated dried pineapple slices.

Sample Condition		L∗	a∗	b∗	Chroma	Hue Angle	WI	BI	Δ*BI*	Δ*E*
Fresh		75.39 ± 1.35^a^	2.03 ± 0.46^b^	26.81 ± 3.96^bc^	26.89 ± 3.98^c^	85.72 ± 0.36^a^	63.42 ± 2.05^a^	44.89 ± 7.33^c^	-	-
Drying at 50 °C	UTR	63.63 ± 3.33^bc^	12.76 ± 0.51^a^	38.68 ± 4.26^a^	38.41 ± 6.04^ab^	72.61 ± 1.95^b^	45.23 ± 1.41^b^	103.36 ± 8.05^ab^	58.46 ± 14.74^ab^	79.18 ± 3.58^a^
	1TR	55.86 ± 6.27^bcd^	10.60 ± 1.85^a^	28.13 ± 0.62^bc^	30.10 ± 1.18^bc^	69.48 ± 2.97^b^	46.47 ± 4.72^b^	82.75 ± 9.12^b^	37.85 ± 6.17^b^	77.84 ± 5.52^a^
	2TR	64.42 ± 1.19^b^	11.19 ± 1.64^a^	39.19 ± 7.69^a^	40.77 ± 7.83^a^	73.91 ± 1.10^b^	45.68 ± 5.22^b^	102.54 ± 25.09^ab^	57.64 ± 17.79^ab^	80.99 ± 3.17^a^
	3TR	55.44 ± 4.06^bcd^	11.76 ± 0.40^a^	33.07 ± 3.36^abc^	35.12 ± 3.18^abc^	70.25 ± 1.93^b^	43.13 ± 1.59^bc^	101.84 ± 4.37^a^	56.95 ± 10.88^ab^	75.92 ± 3.24^a^
	4TR	58.10 ± 3.01^bcd^	10.79 ± 0.55^a^	32.59 ± 2.48^abc^	34.34 ± 2.46^abc^	71.64 ± 1.07^b^	45.75 ± 1.54^b^	92.68 ± 6.35^ab^	47.78 ± 8.88^ab^	79.73 ± 2.31^a^
Drying at 55 °C	UTR	57.53 ± 0.46^bcd^	11.12 ± 0.73^a^	35.39 ± 1.65^ab^	37.12 ± 1.37^ab^	72.50 ± 1.82^b^	43.59 ± 0.82^bc^	104.56 ± 4.91^ab^	59.66 ± 12.01^ab^	77.69 ± 0.64^a^
	1TR	49.48 ± 1.99^de^	12.16 ± 0.28^a^	28.82 ± 1.23^bc^	30.88 ± 1.21^bc^	68.92 ± 0.71^bc^	40.56 ± 2.05^bc^	101.78 ± 9.39^ab^	56.88 ± 4.47^ab^	71.87 ± 3.09^ab^
	2TR	41.91 ± 5.04^e^	12.12 ± 0.09^a^	23.99 ± 2.09^c^	26.72 ± 1.84^c^	63.79 ± 2.21^c^	35.92 ± 3.87^c^	103.15 ± 7.08^ab^	58.25 ± 14.16^ab^	64.85 ± 3.68^b^
	3TR	51.55 ± 0.71^d^	12.34 ± 1.81^a^	33.63 ± 3.82^ab^	34.96 ± 1.91^abc^	68.54 ± 0.76^bc^	39.66 ± 1.95^bc^	116.56 ± 17.28^ab^	71.66 ± 10.42^a^ab	73.13 ± 1.147^ab^
	4TR	51.11 ± 0.39^de^	10.38 ± 0.40^a^	27.01 ± 1.97^bc^	28.94 ± 1.78^bc^	68.88 ± 1.86^bc^	43.18 ± 1.00^bc^	87.68 ± 7.23^ab^	42.78 ± 14.45^ab^	73.89 ± 1.24^ab^
Drying at 60 °C	UTR	58.47 ± 1.87^bcd^	9.70 ± 0.58^a^	32.52 ± 0.89^abc^	33.95 ± 0.78^abc^	73.33 ± 1.27^b^	46.35 ± 1.26^b^	90.21 ± 3.53^ab^	45.31 ± 4.09^ab^	80.22 ± 1.34^a^
	1TR	54.41 ± 2.46^cd^	11.07 ± 0.75^a^	29.89 ± 3.48^abc^	31.93 ± 3.01^abc^	69.39 ± 3.45^b^	44.26 ± 0.67^b^	91.65 ± 6.77^ab^	46.75 ± 4.68^ab^	76.93 ± 1.58^a^
	2TR	53.66 ± 1.11^d^	11.59 ± 0.29^a^	31.80 ± 0.39^abc^	33.85 ± 0.28^abc^	69.95 ± 0.68^b^	42.61 ± 0.85^bc^	101.43 ± 2.69^ab^	56.53 ± 9.99^ab^	75.52 ± 0.79^a^
	3TR	55.29 ± 2.70^bcd^	10.41 ± 1.04^a^	30.81 ± 1.31^abc^	32.52 ± 1.58^abc^	71.37 ± 1.01^b^	44.71 ± 3.11^b^	92.71 ± 12.24^ab^	47.81 ± 4.99^ab^	77.68 ± 3.40^a^
	4TR	54.74 ± 5.69^cd^	11.16 ± 1.41^a^	30.85 ± 0.38^abc^	32.84 ± 0.37^abc^	70.13 ± 2.47^b^	44.03 ± 4.80^bc^	95.85 ± 15.09^ab^	50.95 ± 7.77^ab^	76.60 ± 5.96^a^

-All the values in the table are mean ± SD of three independent determinations. Samples in the same column with different superscript letters differ significantly p < 0.05.

Besides, as drying temperature increased from 50 to 60 °C, shrinkage decreased. It is assumed that at the higher drying temperature, the drying rate increased, and the pineapple surfaces became mechanically stable, resulting in less shrinkage of pineapple slices. Similar results were observed for dried apples by Lewicki and Jakubzcyk (2004). Önal et al. (2019) found similar results for persimmon samples where they found that at 60–65 °C drying temperatures, the shrinkage was lower.

Sturm et al. (2012) also noticed that hot-air dried apples exhibited lower shrinkage with increasing air velocity and temperature. From the experimental data, the volumetric shrinkage coefficient (S_b_) was the lowest 0.21 ± 0.07 for trehalose treated pineapple slices at 55 °C drying temperature and the values of all untreated and treated dried samples ranged from 0.21 ± 0.07 to 0.32 ± 0.01 (Table 3). The statistical analysis (ANOVA) explains that there are no significant differences (p > 0.05) within different values of the volumetric shrinkage coefficient for different samples dried at various temperatures.

### 3.5 Color parameters

Color is one of the essential quality parameters of dried food products for their economic and sensorial value. The lightness value L∗ indicates the darkness/lightness with a range from 0 (black) to 100 (white), a∗ index represents greenness/redness, and b∗ index represents blueness/yellowness of the sample. The L∗-values changed from the range of 75.39 ± 1.35 to 41.91 ± 5.04. As brightness decreased in dried samples, the L∗ values were also reduced compared to the fresh pineapple slices. Usually, the L∗-values reduced with the increasing temperatures. The relative visual red color (a∗-values) and corresponding visible yellow color (b∗-values) increased during drying. However, the b∗-values were increased relatively slightly at a higher temperature (60 °C). Ramallo and Mascheroni (2012) also reported similar a∗ and b∗values in untreated pineapple slices. It is thought that pineapple samples become darker as drying continues due to non-enzymatic browning reactions.

Hue is a crucial analytical tool for describing the color since it determines the redness, yellowness, greenness, blueness, etc., of a product. A pale-yellow color is engaged with ripening and development of the flavor characteristics of pineapple. Consumers use this factor for decision-making in products' acceptability (Bartolome et al., 1996). The hue color parameter showed more significance and changed regularly during drying. The hue angle values differed from 0 (pure red color) to 90 (pure yellow color), 180 (pure green color), and 270 (pure blue color) (Ramallo and Mascheroni, 2012). From the fresh pineapples’ measurements, the value of hue was 85.72 ± 0.36, and this value was lowered during drying. This fact indicates a reduction of yellow color and an increment of red color (hue<90).

Chroma or saturation describes the purity of color. The higher the chroma value, the more pure and intense the color (Ramallo and Mascheroni, 2012). Chroma showed a slight increase during drying. Chroma values ranged from 26.89 ± 3.98 to 40.77 ± 7.83 (Table 4). Ramallo and Mascheroni (2012) reported similar observations about hue angles and chroma values.

There are numerous biochemical and physical factors responsible for the color changes of a product during heat processing. Fruits like pineapples with high polyphenols and polyphenol oxidase (PPO) enzymes are very susceptible to enzymatic browning. The non-enzymatic browning may also happen in dried products due to Maillard reaction, caramelization, etc (Deng et al., 2017). The effects of dipping pre-treatments and drying temperatures on the surface color of fresh and dried samples are shown in Table 5. White index (WI), browning index differences (Δ*BI*), and total color differences (ΔE) were also reported. Experimental results proved that the pre-treatment and drying process had substantial effects on the color parameters of fresh and dried pineapple slices. The enzymatic and non-enzymatic browning significantly reduced the white index (WI) in dried samples compared to fresh ones. Albanese et al. (2007) reported the same for dried apples. The differences in WI in the analyzed range of temperatures were not that significant among all the samples. For sensory appeal, the samples with low values of color changes (ΔE) are considered the best since these low values of ΔE indicate little change of color from the actual.

**Table 5:** Microbial load of pineapple samples.

Sample Conditions		Microbial Load	
		Fungal Count CFU/g	Bacterial Count CFU/g
Fresh		2.56 ×$10^{5}\pm 8.54$^a^	5.34 ×$10^{6}\pm 10.02$^a^
Drying at 50 °C	UTR	3.27 ×$10^{4}\pm 3.51$^b^	6.03 ×$10^{5}\pm 3.06$^b^
	1TR	2.73 ×$10^{3}\pm 4.51$^cd^	6.37 ×$10^{4}\pm 3.51$^c^
	2TR	1.15 ×$10^{3}\pm 3.00$^g^	2.50 ×$10^{4}\pm 2.65$^ef^
	3TR	1.62 ×$10^{3}\pm 2.52$^efg^	4.47 ×$10^{4}\pm 2.52$^d^
	4TR	1.72 ×$10^{3}\pm 1.53$^efg^	1.90 ×$10^{4}\pm 2.00$^ef^
Drying at 55 °C	UTR	1.85 ×$10^{4}\pm 7.62$^b^	2.10 ×$10^{5}\pm 2.00$^b^
	1TR	2.05 ×$10^{3}\pm 2.00$^def^	2.43 ×$10^{4}\pm 2.08$^ef^
	2TR	4.10 ×$10^{3}\pm 3.00$^b^	2.90 ×$10^{4}\pm 4.58$^e^
	3TR	2.08 ×$10^{3}\pm 3.51$^de^	1.37 ×$10^{4}\pm 3.51$^f^
	4TR	1.23 ×$10^{3}\pm 3.06$^g^	2.27 ×$10^{4}\pm 1.53$^ef^
Drying at 60 °C	UTR	3.28 ×$10^{4}\pm 3.51$^b^	7.80 ×$10^{5}\pm 2.00$^b^
	1TR	1.82 ×$10^{3}\pm 4.04$^efg^	4.60 ×$10^{4}\pm 2.00$^d^
	2TR	2.13 ×$10^{3}\pm 4.04$^de^	5.47 ×$10^{4}\pm 6.06$^cd^
	3TR	1.25 ×$10^{3}\pm 3.00$^fg^	2.37 ×$10^{4}\pm 3.51$^ef^
	4TR	1.33 ×$10^{3}\pm 2.52$^fg^	2.67 ×$10^{4}\pm 4.04$^ef^

-All the values in the table are mean ± SD of three independent determinations. Samples in the same column with different superscript letters differ significantly at p < 0.05.

As expected, the smaller amounts of ΔE were found in pre-treated samples dried at 55 °C (Table 5). According to the study of Vega-Gálvez et al. (2012), a short drying time at a higher temperature is associated with the lesser discoloration of apple than an extended drying time due to the formation of browning products by simultaneous heat and mass transfer. However, the enzymatic browning reaction was not studied as browning enzymes are usually prone to degrade at more than 50 °C (Martinez and Whitaker, 1995). The highest Δ*BI* (71.66 ± 10.42) obtained for the sucrose treated sample at 55 °C, and the lowest (37.85 ± 6.17) was for the trehalose treated sample at 50 °C Hosseinpour et al. (2013) reported that the escalated Δ*BI* gave rise to the brown coloration of the specimens developed by the destruction of color pigments, Maillard reaction, and shrinkage of the sample during drying.

### 3.6 Microbial load

The microbial load (Fungal count and Bacterial count) of fresh, untreated, and treated dried pineapple samples were demonstrated. The fungal and bacterial count of unprocessed pineapple samples were 2.56 ×$\;10^{5}$ and 5.34 ×$10^{6}$ (CFU/g), respectively. After drying, the microbial loads of treated and untreated pineapple samples were reduced for the fungal and bacterial count. The microbial load of pre-treated and untreated dried samples decreased by 2 logs and 1 log, respectively, compared to the fresh samples. The sucrose pre-treatment was shown a lower count of both bacterial and fungal count. Mitrakas et al. (2008) reported that 1.67 log CFU/cm^2^ reduction of mesophilic bacteria for potato slices dried at 40 °C with 50% sucrose pre-treatment. There is a chance of further recontamination of the sample during the earlier stage of the drying process. Fortunately, when the moisture content of dried product comes under 10–12%, the chance of growth of microorganisms becomes lower as the samples' water activity become lower and free water in the products become unavailable for microbes (Bourdoux et al., 2016). Zapata et al. (2011) found a reduction of more than 2 logs in microbial counts of dehydrated pineapples when treated with 2.48% citric acid and dried at 44.99 °C. According to the guideline of Indian microbiological standards for dried fruits and vegetables and their products, the satisfactory aerobic plate count level of dried fruits is 4 × 10^4^ CFU/g and acceptable below 1 × 10^5^ CFU/g. Besides, the satisfactory fungal load in dried fruits is 1 × 10^2^ CFU/g and acceptable below 1×10^4^ (FSSAI, 2018). Ireland's guidelines for the microbiological quality at the point of sale suggested that <10^5^ CFU/g level is satisfactory for dried fruits (Scientific Criteria to Ensure Safe Food, 2003). The present study results reveal that most of the dried pineapple slices except untreated one meet the above mentioned microbiological safety standards. The microbial count in the fresh and dehydrated pineapple samples are presented in Table 5.

### 3.7 Drying time

**Figure 3:**
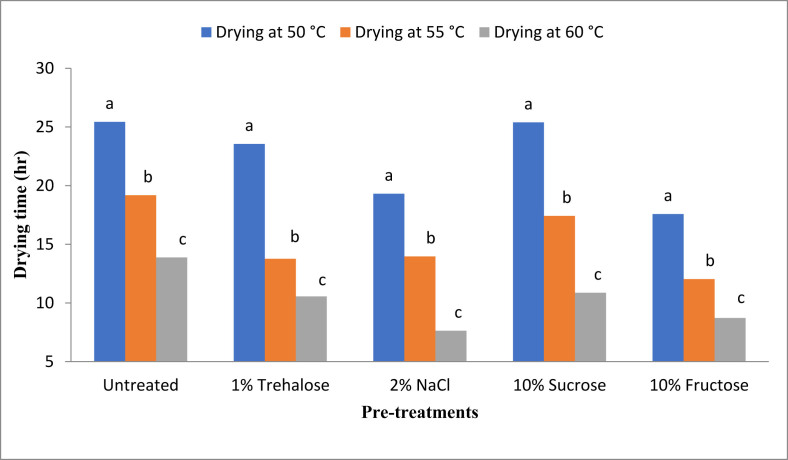
Drying time required to reach 20% moisture content (wet basis) by different pinapple slices.

Drying time for all specimens was estimated to know which experimental treatments required the least dehydration time. The lowest drying time required was 7.64 h using 2% NaCl pre-treatment at 60 °C, considering time to reach 20% moisture content in the dried product at 30% RH. On the other hand, the longest drying time was 25.44 h for the untreated sample at 50 °C. When pre-treated with 2% NaCl, the pineapple samples presented the lowest drying time compared to other pre-treatments using the same processing conditions. NaCl pre-treated samples needed 44.96% lower drying time than untreated samples, considering 65 °C drying temperature. At 60 and 55 °C, the lowest drying time was 12.04 h and 17.58 h, respectively, required by fructose pre-treated samples. It was observed that a 10 unit increase in temperature reduced the drying time to almost half. The results of this study are in line with the findings of previous studies where researchers showed that a 56.9%, 49.5%, and 25% reduction in drying time was possible for melon, pumpkin, and kiwifruit, respectively (Önal et al., 2019; Rojas and Augusto 2018; Wang et al., 2019). The drying time required to reach 20% moisture content (wet basis) by different pineapple slices has been represented in Figure 3.

During drying, the of dried products’ physical parameters play crucial roles as it directly related to consumers' acceptability. In this study, different physical parameters like-changes in Color parameters, White index, Browning index, Coefficient of Rehydration, Rehydration Ratio, and Volumetric shrinkage coefficient, etc., were studied. Besides, pineapples are prosperous sources of vitamins (vitamin A, thiamine, vitamin B_6_, folate, vitamin C, etc.), and antioxidants (Bartolomé et al., 1995; Ramallo and Mascheroni, 2012). As B vitamins and vitamin C are both water-soluble as well as high heat-sensitive, an attempt was made to minimize the losses of these nutrients during pre-treatments and drying. Additionally, drying time is also a significant parameter for efficient drying; hence this should also be one of the prime concerns.

Consequently, to conclude the best pre-treatments and drying temperatures of this study, we need to consider changes in both the physical and biochemical parameters. Starting with the physical parameters, pineapple slices dried at 55 °C using 1% trehalose pre-treatment (1TR) showed the best results considering Coefficient of Rehydration, Rehydration Ratio, and Volumetric shrinkage coefficient, Color parameters, White index, Browning index, etc. At this processing condition, there were some insignificant reductions in biochemical properties like vitamins and antioxidant activity of dried samples, such as total Phenolic Content (TPC), Total Flavonoid Content (TFC), and DPPH radical scavenging assay. However, pineapple slabs with 10% fructose pre-treatments (4TR) showed the best retention of most of the biochemical properties among all the samples at 60 °C drying temperature. This condition showed the best retention of vitamins, antioxidants, acidity, TSS, and considerable microbial load, which might be due to the shortest drying times at elevated temperatures (60 °C). The untreated samples also showed excellent results in biochemical properties, but they need the longest time for drying, which is associated with massive energy consumption and time; and results in inferior physical properties of dried samples. Besides, the untreated dried pineapple slices exhibited microbial load above the acceptable safety limits. In a nutshell, drying at 55 °C using 1% trehalose pre-treatment (1TR) exhibited the best physical parameters with some insignificant loss in biochemical parameters, and drying at 60 °C using 10% fructose pre-treatment (4TR) represented better biochemical quality retentions with minimum drying time. Considering all the physical and biochemical properties and drying time, we can propose that drying at 55 °C using 1% trehalose pre-treatment (1TR) is the best among different drying conditions.

## 4 Conclusions

Both pre-treatments and temperature influenced the drying process and the quality characteristics of the pineapple slabs. Moreover, the optimal drying temperature combined with pre-treatments was 55 °C drying temperature for 30% RH, which preserved the best physical quality of the pineapple slices in shorter drying time. The best product condition was retained at 55 °C drying temperature with 1% trehalose pre-treatment, which was exihibited by the lowest volumetric shrinkage coefficient (0.21), and the highest rehydration ratio (6.840) and Coefficient of Rehydration (0.941). It also sustained the physicochemical properties, nutritional quality, the lower color changes, lower microbial load, lower shrinkage, finer structure preservation, and better rehydration capability. Although the quantity of vitamin C, and antioxidant activity of the samples were slightly decreased by increasing drying temperature, the ascorbic acid and antioxidant activity of the dried samples preserved by the pre-treatment process. This study suggests that higher dehydration temperatures reduce the drying time considerably, which help to retain the physico-chemical properties of pineapple. Our study will help future researchers to develop novel preservation technique by optimizing immersion pre-treatment and drying temperatures for shelf life extension of perishable items like fruits and vegetables. Optimization of drying conditions by maintaining variables such as pre-treatments, temperature, relative humidity, and drying time could be an area of interest for further research.

## Declarations

### Author contribution statement

Wahidu Zzaman: Analyzed and interpreted the data; Contributed reagents, materials, analysis tools or data; Wrote the paper.

Rahul Biswas: Performed the experiments; Analyzed and interpreted the data; Wrote the paper.

Mohammad Afzal Hossain: Conceived and designed the experiments; Analyzed and interpreted the data; Contributed reagents, materials, analysis tools or data; Wrote the paper.

### Funding statement

This work was supported by the University Research Centre, 10.13039/501100007944Shahjalal University of Science and Technology, Sylhet-3114, Bangladesh (AS/2019/1/35).

### Data availability statement

Data included in article/supplementary material/referenced in article.

### Declaration of interests statement

The authors declare no conflict of interest.

### Additional information

No additional information is available for this paper.
